# Integration of transcriptome and phytohormone analyses reveals antagonistic interactions between jasmonate and auxin signaling pathways in rice (*Oryza sativa* L.)

**DOI:** 10.3389/fpls.2025.1622785

**Published:** 2025-09-17

**Authors:** Xiaoying Wu, Yuting Cao, Yeye Chen, Wei Xiong, Gaorong Cao, Yan Zeng, Liqin Zhang, Rensen Zeng, Dongmei Chen

**Affiliations:** ^1^ Zhejiang Key Laboratory of Biology and Ecological Regulation of Crop Pathogens and Insects, College of Life Science, Huzhou University, Huzhou, Zhejiang, China; ^2^ Key Laboratory of Vector Biology and Pathogen Control of Zhejiang Province, College of Life Science, Huzhou University, Huzhou, Zhejiang, China; ^3^ College of Agriculture, Fujian Agriculture and Forestry University, Fuzhou, China

**Keywords:** jasmonate signaling, auxin, indole-3-acetic acid, transcriptome, antagonistic interaction, hormone crosstalk

## Abstract

Interactions between different hormones plays a central role in plant trade-off between growth and defense. Auxin is a pivotal growth hormone and jasmonates (JA) are key hormones for plant responses to environmental stressors. In this study we examined the interactions between auxin and JA in rice (*Oryza sativa* L.) by integration of transcriptome and phytohormone analyses. Exogenous application of methyl jasmonate (MeJA) to rice leaves led to markedly increased accumulation of jasmonic acid (JA), MeJA, and dihydrojasmonic acid (DJA) in both leaves and roots, as well as N-jasmonic acid isoleucine (JA-Ile) in the leaves. Importantly, MeJA application significantly influenced auxin biosynthesis and orchestrated large-scale changes in transcriptional regulation of L-tryptophan (Trp) biosynthesis, as well as indole-3-acetic acid (IAA) biosynthesis, catabolism, signaling transduction, and transport in rice leaves and roots, leading to notably decreased IAA in the leaves and roots, but increased levels of indole-3-carboxylic acid (ICA) and indole-3-carboxaldehyde (ICAld) in the leaves. Our findings suggest that JA signaling suppresses auxin signaling by reducing Trp flux into the indole-3-pyruvic acid (IPA) pathway, while enhancing Trp flux into the tryptamine (TAM) pathway, thereby fine-tuning rice growth upon JA burst.

## 1 Introduction

In order to survive in natural environments, plants must effectively defend against a diverse array of stressors, encompassing both biotic and abiotic challenges. However, this defense incurs substantial costs and is frequently associated with considerable growth inhibition (Huot et al., 2014; Li et al., 2015). Throughout the course of evolution, plants have developed intricate mechanisms to maximize protection against stressors while minimizing deviations from optimal growth and fitness through a delicate interplay among phytohormones (Meldau et al., 2012; Machado et al., 2013). Jasmonates (JAs), encompassing jasmonic acid (JA) and its derivatives, function as stress-associated phytohormones that are integral to plant defense mechanisms against herbivores and pathogens, as well as to the tolerance of abiotic stress and the regulation of plant development, such as flower development and fertility (Moreno et al., 2009; Wasternack and Hause, 2013; Wasternack and Song, 2017; Han, 2017; Huang et al., 2017; Li et al., 2022). The activation of JAs-mediated defense signaling typically imposes constraints on plant growth to improve plant tolerance and resilience during tough times (Moreno et al., 2009; Yang et al., 2012; Li et al., 2015; Rivero et al., 2022; Hewedy et al., 2023), exemplifying the classical trade-off between defense and growth in plants. Although molecular mechanisms governing JA-mediated growth regulation under adverse stress conditions continue to be actively investigated, it is evident that these processes are predominantly modulated through cross-talk with other hormones, notably auxin.

Auxin, represented by indole-3-acetic acid (IAA), is a vital growth hormone central to nearly all aspects of plant growth and development, and environmental adaptation (Woodward and Bartel, 2005; Enders and Strader, 2015; Gomes and Scortecci, 2021). To ensure optimal plant growth under fluctuating environmental conditions, it is imperative that local auxin concentrations and activities are meticulously regulated. This regulation encompasses the processes of auxin biosynthesis, signal transduction, transport, degradation, and the interconversion between its active and inactive forms, etc (Zhao, 2018; Mazzoni-Putman et al., 2021; Casanova-Sáez et al., 2021). Over the past several decades, despite extensive research on auxin’s roles in plant development, the molecular mechanisms governing its stress response dynamics–particularly regarding crosstalk with JA signaling–continue to be actively defined.

Researchers have identified numerous instances of interaction between JAs and auxin signaling pathways, which may occur at the levels of hormone perception, gene expression, or the modulation of each other’s homeostasis and transport (Pérez and Goossens, 2013; Zhang et al., 2016; Mazzoni-Putman et al., 2021). Evidence indicates that auxin is rapidly induced following herbivore attacks and subsequently regulates JA-dependent defense pathways (MaChado et al., 2016). The JA signaling pathway is linked to auxin homeostasis through the modulation of the expression of IAA biosynthetic genes, including *ANTHRANILATE SYNTHASE ASA1/ASB1* and certain members of the *YUCCA* family (Sun et al., 2009; Hentrich et al., 2013; Cai et al., 2014). Furthermore, jasmonate has been documented to influence the distribution of the auxin exporter PIN2, thereby modulating auxin transport (Sun et al., 2011). Conversely, AUXIN RESPONSE FACTOR ARF6 and ARF8 play a crucial role in flower development, partly by activating JA synthesis (Nagpal et al., 2005; Tabata et al., 2010). Auxin-inducible acyl amidosynthetases, specifically GRETCHEN HAGEN3 (GH3.3, GH3.5, and GH3.6), modulate JA homeostasis to initiate the formation of adventitious roots (Gutierrez et al., 2012). Moreover, in response to tissue damage, JA and auxin signaling exhibit a synergistic interaction that activates the ERF115 transcription factor, a pivotal factor in tissue regeneration (Zhou et al., 2019; Zhang et al., 2023). Recently, JA-mediated MYB Transcription Factor 1 (JMTF1) was reported as a coordinator in maintaining the balance between JA and auxin signaling in rice defense response (Uji et al., 2024).

Physiological and genetic studies have undoubtedly underscored the significant interaction between JAs and auxin pathways in plant growth and stress adaptation. Nonetheless, the comprehension of this crosstalk remains puzzle and fragmented. Most evidences of hormone interactions are obtained in the model plant *Arabidopsis*. In this study, we employed a combination of comparative transcriptome analysis and endogenous hormone profiling to elucidate the relationship between JAs and auxin rice (*Oryza sativa* L.), a staple food crop. Our aim was to provide a comprehensive overview of auxin biosynthesis and signal transduction dynamics as modulated by JA signaling in rice.

## 2 Materials and method

### 2.1 Plant material and culture

In this study, rice (*Oryza sativa* L. cv. Shishoubaimao) seeds were surface sterilized with 10% H_2_O_2_ for 10 minutes, rinsed thrice with distilled water, and pre-soaked for a day. The seeds were pre-germinated at 28°C for 2 days, then placed in culture dishes with 0.5× modified Kimura B solution in a growth chamber for 7 days. Finally, seedlings were transplanted into plastic boxes with 1× modified Kimura B solution in a greenhouse. Rice plants were grown at 30°C/26°C (day/night temperatures), 75% humidity, with natural daylight, and nutrient solutions were refreshed every three days using a 1×modified Kimura B solution per Wu et al. (2019).

### 2.2 Methyl jasmonate and auxin homolog treatment

After 21 days in a greenhouse, uniformly sized rice plants were used for treatment. Rice leaves were sprayed with a 500 μM MeJA (Sigma-Aldrich, USA) solution with 0.01% Tween 20, while control plants received only 0.01% Tween 20. The use of a 500 µM concentration for foliar application is scientifically justified, given MeJA’s volatility, limited foliar uptake, and demonstrated efficacy, aligning with established methodologies in plant JA studies (Gómez et al., 2010; Pak et al., 2021). Our prior work confirmed that 500 µM MeJA effectively activates JA signaling and downstream responses (Wu et al., 2019), in addition to causing observable growth inhibition (**Supplementary Figure S1**). Both sets of plants were enclosed in transparent boxes respectively to ensure solution absorption. Leaves and roots were sampled from both groups at 3, 6, and 12 hours post-treatment, quickly frozen in liquid nitrogen, and stored at -80°C for subsequent transcriptome and hormone analyses. In addition, rice leaves were harvested four days post-treatment for L-tryptophan (Trp) determination, and MeJA was applied once daily during the four-day treatment period.

In the MeJA and IAA combination study, rice plants were cultivated in a 1× modified Kimura B solution, with or without the addition of 0.1 μM IAA (Sangon Biotech, China). Concurrently, the rice leaves were sprayed with or without 500 μM MeJA solution, as previously described. Finally, four distinct treatments included: control group (without IAA and MeJA), MeJA group (treated with MeJA), IAA group (treated with IAA), and IAA+MeJA group (treated with both IAA and MeJA). The leaves were harvested from each treatment group within 12 hours post-treatment, rapidly frozen in liquid nitrogen, and subsequently stored at -80°C for quantitative real-time PCR analysis. Seven days following the application of MeJA and/or IAA treatments, the length, weight, and chlorophyll content of the rice plants were measured. Nutrient solutions were replenished daily, and hormone treatments were administered once per day throughout the 7-day treatment period.

### 2.3 Transcriptome analysis

Total RNA was extracted from leaves and roots using RNAPREP PURE Polysaccharide and Polyphenol Plant Total RNA Extraction Kit (DP441, TIANGEN, China), following the manufacturer’s guidelines. Three biological replicates per treatment were used for transcription analysis. RNA integrity was checked with the RNA Nano 6000 Assay Kit on the Bioanalyzer 2100 system (Agilent Technologies, CA, USA). Novogene Co., Ltd. (Beijing, China) conducted the transcriptome sequencing. Briefly, mRNA was isolated from 1 μg of RNA per sample using poly-T oligo-attached magnetic beads, and the sequencing library was constructed according to the standard procedure. The quality of the library was evaluated using the Qubit 2.0 Fluorometer and the Agilent Bioanalyzer 2100 system. Sequencing was subsequently performed on an Illumina NovaSeq platform, to generate 150 bp paired-end reads. Clean data were obtained by removing reads containing adapters, poly-N sequences, and low-quality reads from the raw data. An index of the reference genome was built, and paired-end clean reads were aligned to the reference genome using HISAT2 (version 2.0.5). Read counts mapped to each gene were determined using featureCounts (version 1.5.0-p3). Subsequently, the FPKM (Fragments Per Kilobase of transcript per Million mapped reads) of each gene was calculated based on the gene length and the corresponding read counts. Differentially expressed genes (DEGs) were identified using the DESeq2 R package (version 1.20.0), with criteria set as |log2 (Fold Change)| > 0 and an adjusted *P*-value < 0.05. The functional characterization of the DEGs was conducted through Gene Ontology (GO) enrichment analysis and Kyoto Encyclopedia of Genes and Genomes (KEGG) pathway enrichment analysis, utilizing the clusterProfiler R package (version 3.4.4). The quality control and mapping information were listed in **Supplementary Table S1**. The results indicated that the sequencing quality was sufficient to support further analysis. All raw sequences have been deposited in the NCBI Sequence Read Archive (SRA) under the accession number PRJNA1299722.

### 2.4 Phytohormone analysis

Five IAA-related compounds, 3-indolebutyric acid (IBA), indole-3-carboxylic acid (ICA), indole-3-carboxaldehyde (ICAld), and methyl indole-3-acetate (MeIAA)), and four JAs (JA, MeJA, N-jasmonic acid isoleucine (JA-Ile), and dihydrojasmonic acid (DJA)) were detected in this study. The tissue samples were ground with liquid nitrogen, after which 100 mg of the resulting powder was weighed, and 400 μL of a solution containing a mixed internal standard (50% acetonitrile) was added. The mixture was then vortexed thoroughly and extracted at 4°C. The supernatant collected via centrifugation was initially passed through the HLB sorbent, resulting in the first flow-through fraction. Subsequently, it was eluted with 500 μL of 30% acetonitrile, yielding the second flow-through fraction. These two fractions were then combined in a single centrifuge tube and thoroughly mixed. The resulting solutions were used to quantitate phytohormone by an ultra-high performance liquid chromatography coupled with tandem mass spectrometry (UHPLC-MS/MS) system (ExionLC™ AD UHPLC-QTRAP 6500+, AB SCIEX Corp., Boston, MA, USA), at Novogene Co., Ltd (Beijing, China). Chromatographic separation was conducted using a Waters XSelect HSS T3 column (2.1 × 150mm, 2.5 μm) maintained at a temperature of 45°C. The mobile phase comprised 0.01% formic acid in water (solvent A) and 0.01% formic acid in acetonitrile (solvent B), delivered at a flow rate of 0.30 mL/min. The solvent gradient was programmed as follows: initial 10% B, 1min; 10-50% B, 3min; 50-65% B, 4min; 65-70% B, 6min; 70-100% B, 7min; 100-10% B, 9.1min; 10% B, 12min. The mass spectrometer was operated in multiple reaction monitoring (MRM) mode, with the following parameters: IonSpray voltage set at -4500V for negative mode and 4500V for positive mode, curtain gas pressure maintained at 35psi, ion source temperature at 550°C, and ion source gases 1 and 2 both set at 60psi. Hormone analysis was conducted using four biological replicates per treatment. All phytohormone standards and stable isotope-labeled standards were procured from ZZ Standards Co., Ltd (Shanghai, China). Catalog numbers of phytohormone standards are provided in **Supporting Information Table S2**.

### 2.5 Trp and chlorophyll content determination

Leaf samples (0.2g) were ground using liquid nitrogen, followed by the addition of 2ml of 6% sulfosalicylic acid to facilitate amino acid extraction. The extract was subsequently filtered through a 0.22 μm membrane filter, and the Trp content was quantified utilizing an L-8800 amino acid analyzer (Hitachi, Japan). Chlorophyll was extracted from the leaf samples using 95% ethanol, and its concentration was measured at wavelengths of 665 nm and 649 nm, as described by Wu et al. (2019).

### 2.6 Quantitative real-time PCR analysis

Total RNA was extracted from 0.1g samples utilizing the MiniBEST Plant RNA Extraction Kit (TaKaRa, Japan) in accordance with the manufacturer’s protocol. Subsequently, first-strand cDNA synthesis was conducted using the PrimeScript™ RT Master Mix (Perfect Real Time, TaKaRa, Japan) following the manufacturer’s guidelines. Quantitative real-time PCR was carried out employing the TB Green Premix Ex Taq™ II (Tli RNaseH Plus, TaKaRa, Japan) on a CFX Connect fluorescent quantitative PCR detection system (Bio-Rad, USA). The thermal cycling conditions were as follows: initial denaturation at 95°C for 30 s, followed by 40 cycles of 95°C for 15 s, 60°C for 30 s, and 72°C for 30 s. Melt curve analysis and agarose gel electrophoresis were performed to confirm the specificity of the amplicons. Relative transcript levels were determined employing the double-standard curve method, with the rice housekeeping gene *OsActin* serving as an endogenous control. The sequences of the gene-specific primers utilized in this study are detailed in **Supplementary Table S3**. All assays were conducted with three biological replicates for each treatment.

### 2.7 Data analysis

The data pertaining to JAs content, IAA and IAA-related compounds content, chlorophyll content, as well as the length and weight of rice plants, and the expression of chlorophyll-related genes were analyzed using factorial ANOVA followed by a Tukey *post-hoc* test (*p* ≤ 0.05). The Trp content data were assessed using a Student’s t-test. All statistical analyses were conducted using the SPSS Statistics software (version 17.0).

## 3 Results

### 3.1 Changes in JAs content

**Figure 1:**
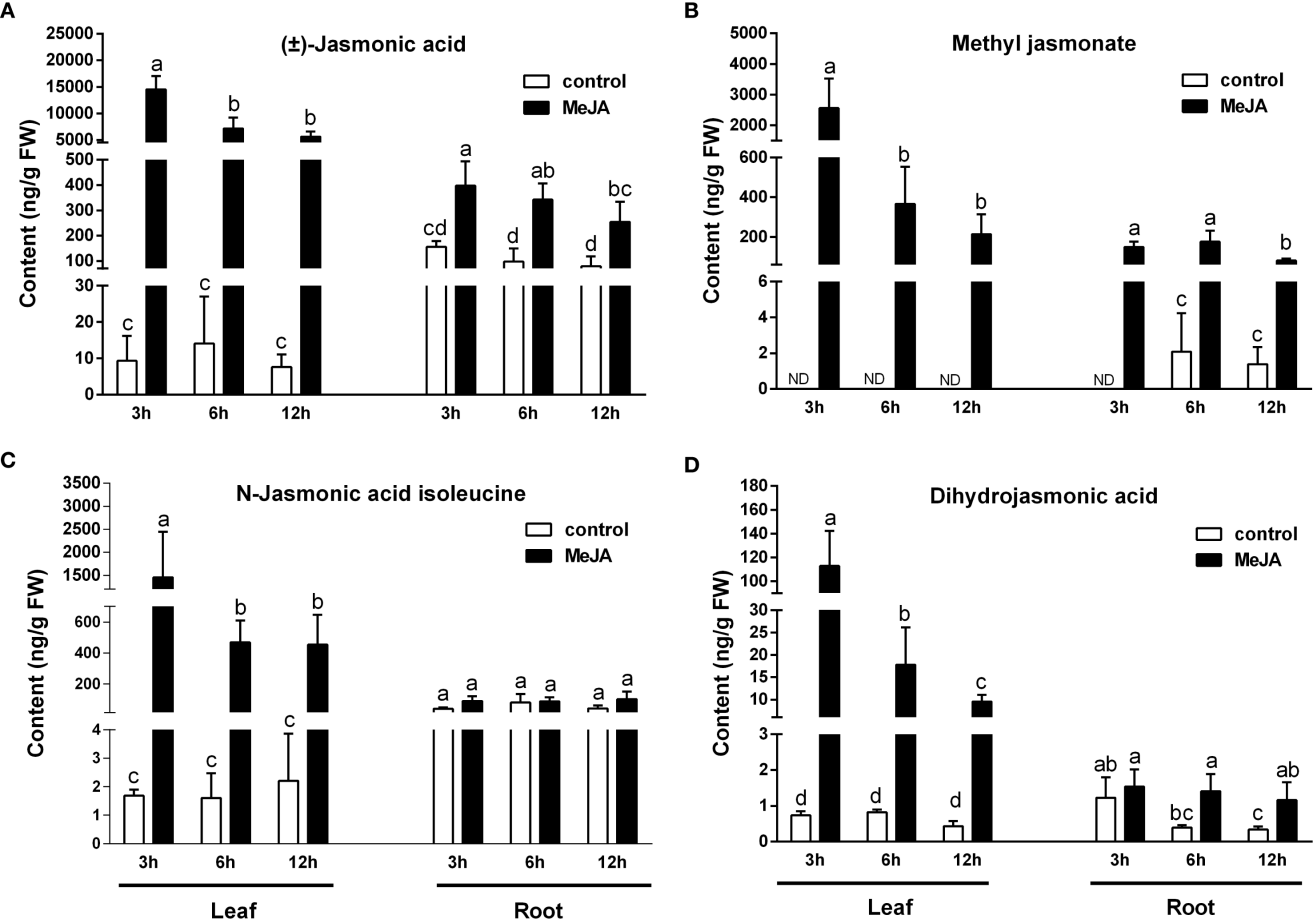
Effects of exogenous MeJA on the content of JAs in the leaves and roots of rice plants. **(A)** (±)-Jasmonic acid. **(B)** Methyl jasmonate. **(C)** N-jasmonic acid isoleucine. **(D)** Dihydrojasmonic acid. Values are mean ± standard SD (n = 4). Significant differences among treatments are indicated by letters above the bars (Tukey's multiple range test, *p* < 0.05).

The exogenous application of MeJA strongly enhanced the accumulation of JA, MeJA, JA-Ile, and DJA in the leaves of rice plants (**Figure 1**). Within 12 hours of MeJA treatment, the levels of JA, JA-Ile, and DJA in the leaves were increased by 734- to 1560-fold, 205- to 864-fold, and 21- to 154-fold, respectively. Initially undetectable in the rice leaves, MeJA concentrations increased significantly to 2560, 367, and 214 ng/g fresh weight at 3, 6, and 12 hr post-treatment, respectively. MeJA treatment also significantly increased the concentrations of JA, MeJA, and DJA in the roots, although the effect was less pronounced compared to that observed in the leaves (**Figure 1**). However, the JA-Ile content in the roots remained unchanged following MeJA treatment.

### 3.2 Changes in expression profiling of Trp biosynthesis related genes

**Figure 2:**
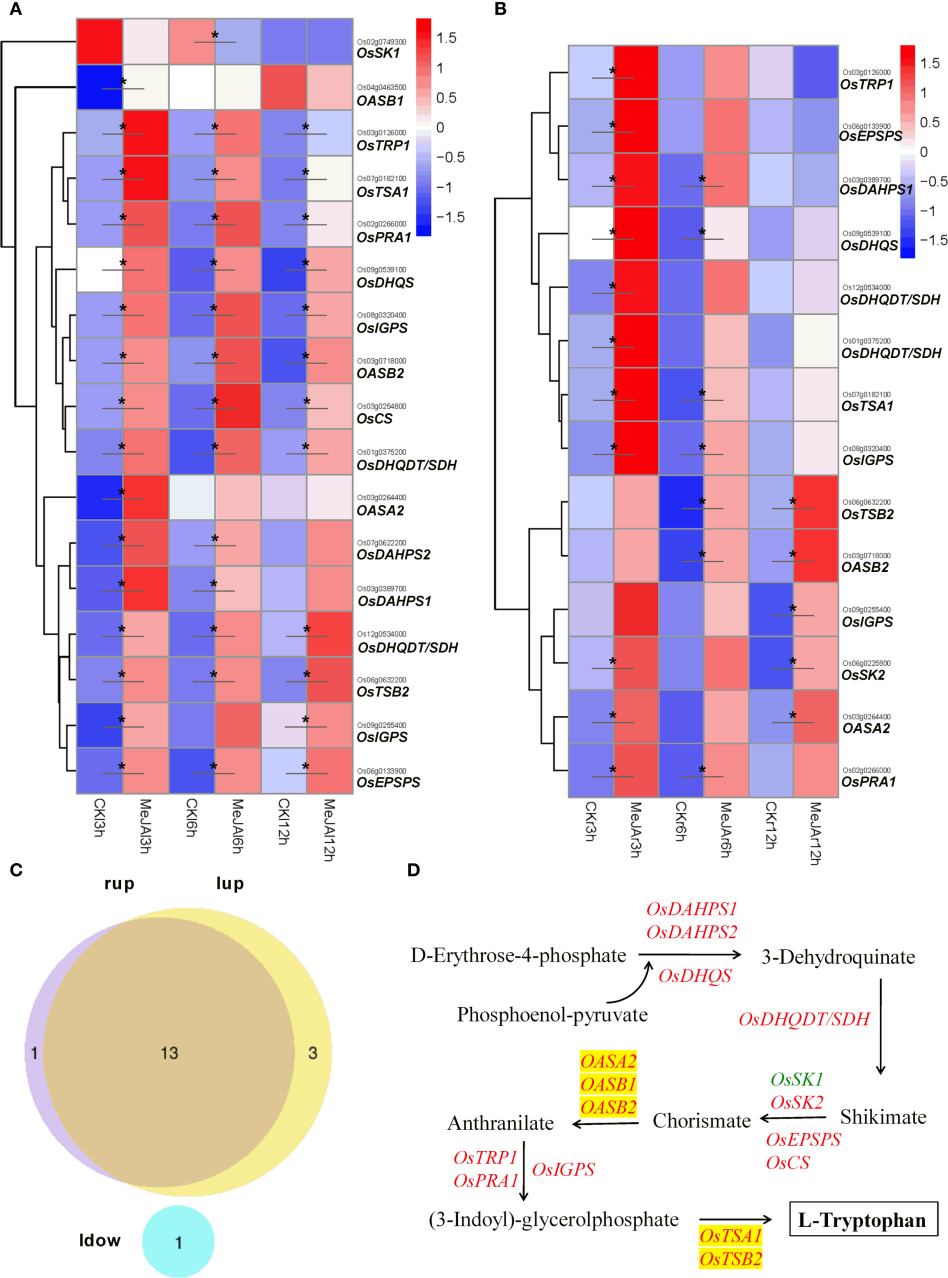
RNA-seq showing effect of exogenous MeJA on the expression of genes associated with Trp biosynthesis in the leaves and roots of rice plants. Hierarchical clustering was performed on differentially expressed genes (DEGs) involved in Trp synthesis, utilizing FPKM values from both MeJA-treated and untreated rice leaves **(A)** and roots **(B)**. CK, Control group. **(C)** Venn diagram that illustrates the overlap of up-regulated and down-regulated DEGs in the leaves and roots of MeJA-treated rice. The terms ‘lup’ and ‘rup’ refer to up-regulated genes in leaves and roots, respectively, while ‘ldown’ denotes down-regulated genes in leaves. **(D)** DEGs within the Trp synthesis pathway, with red and green markers indicating up-regulated and down-regulated genes in the leaves or roots of MeJA-treated rice, respectively. Data were obtained from three biological replicates. DEGs were identified based on a fold change > 1.5 compared to the control, with a q-value (p-adjusted) < 0.05 (marked with *).

As a central precursor for IAA biosynthesis, Trp is synthesized in the chloroplast through the shikimate pathway, a metabolic route by which plants produce aromatic amino acids (Maeda and Dudareva, 2012; **Figure 2D**). Transcriptome sequencing revealed significant changes in the expression of 18 Trp biosynthesis-associated genes across tissues within 12 hours post-MeJA treatment (**Figure 2**). The analysis demonstrated tissue-specific dynamics in Trp biosynthesis genes, with 16, 13, and 12 genes significantly up-regulated in leaves at 3, 6, and 12 hours post-MeJA treatment, respectively. In contrast, roots exhibited up-regulation of 11, 7, and 5 genes at the corresponding time points. Notably, the expression of thirteen genes encoding phospho-2-dehydro-3-deoxyheptonate aldolase (OsDAHPS1), 3-dehydroquinate synthase (OsDHQS), bifunctional 3-dehydroquinate dehydratase/shikimate dehydrogenase (OsDHQDT/SDH), 3-phosphoshikimate 1-carboxyvinyltransferase (OsEPSPS), anthranilate synthase α-subunit (OASA2), anthranilate synthase β-Subunit (OASB2), anthranilate phosphoribosyltransferase (OsTRP1), N-(5’-phosphoribosyl)anthranilate isomerase(OsPRA1), indole-3-glycerol phosphate synthase (OsIGPS), tryptophan synthase alpha chain (OsTSA1), and tryptophan synthase beta chain 2 (OsTSB2) were significantly up-regulated in both leaves and roots. Additionally, the expression of three genes encoding phospho-2-dehydro-3-deoxyheptonate aldolase (OsDAHPS2), chorismate synthase 2 (OsCS) and anthranilate synthase β-Subunit (OASB1) was significantly up-regulated exclusively in leaves, whereas the expression of gene encoding shikimate kinase2 (OsSK2) was significantly up-regulated solely in roots. The expression of the gene encoding shikimate kinase1 (OsSK1) was markedly reduced in the leaves at 6 hr following MeJA treatment.

**Figure 3:**
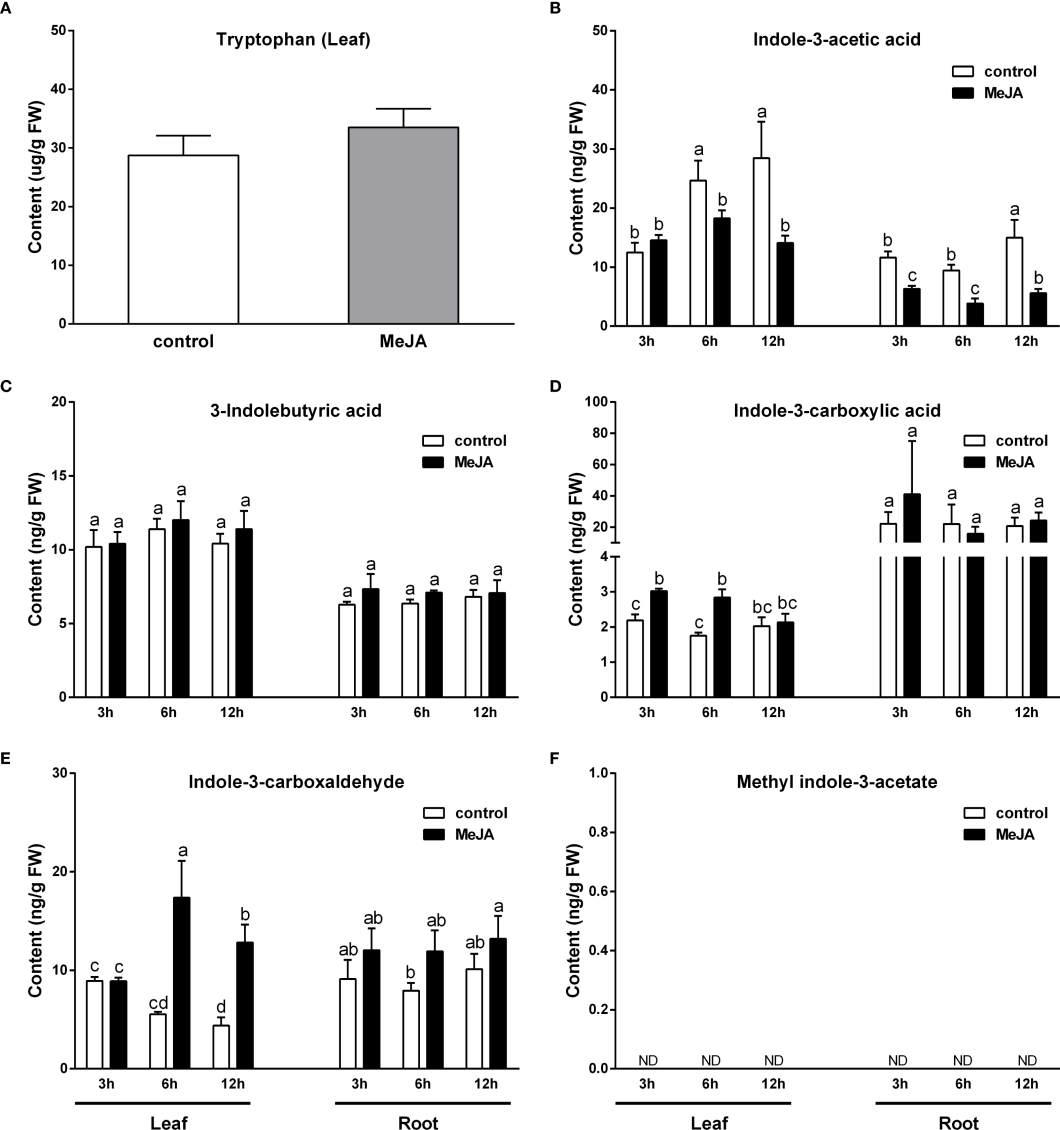
Effect of exogenous MeJA on tryptophan and auxin levels in the leaves and roots of rice plants. **(A)** Tryptophan (Leaf). **(B)** Indole-3-acetic acid. **(C)** 3-Indolebutyric acid. **(D)** Indole-3-carboxylic acid. **(E)** Indole-3-carboxaldehyde. **(F)** Methyl indole-3-acetate. Values are mean ± standard SD (n = 4). Significant differences among treatments are indicated by letters above the bars (Tukey's multiple range test, *p* < 0.05).

Meanwhile, after four days of MeJA exposure, the leaves treated with MeJA did not exhibit a significant accumulation of Trp, suggesting that Trp was rapidly converted (**Figure 3A**).

### 3.3 Changes in IAA and IAA-related compounds content

Following a 3-hr exposure to MeJA, a significant reduction in IAA content was observed in rice roots; subsequently, after 6 hours of treatment, a notable decrease in IAA content was also detected in the leaves (**Figure 3B**). Conversely, the levels of ICA and CAld in the leaves exhibited significant increases at 3 to 6 hr and 6 to 12 hr post-MeJA treatment, respectively (**Figures 3D, E**). In the roots, MeJA treatment did not result in a significant alteration of ICA and ICAld levels, although a slight increase in ICAld levels was observed. The levels of IBA in both leaves and roots remained unaffected by MeJA treatment (**Figure 3C**), and MeIAA was not detected in either leaves or roots of rice plants (**Figure 3F**).

### 3.4 Changes in expression profiling of auxin biosynthesis/catabolism related genes

Auxin biosynthesis in plants is proposed to occur via two distinct pathways: the tryptophan (Trp)-dependent and the Trp-independent pathways, with limited understanding of the latter (Cao et al., 2019). The Trp-dependent pathway consists of several alternative routes for auxin production, including: (i) the indole-3-pyruvic acid (IPA) pathway, (ii) the tryptamine (TAM) pathway, (iii) the indole-3-acetaldoxime (IAOx) pathway, and (iv) the indole-3-acetamide (IAM) pathway. The IPA pathway has been identified as the primary and essential route for auxin biosynthesis in plants (Casanova-Sáez et al., 2021).

**Figure 4:**
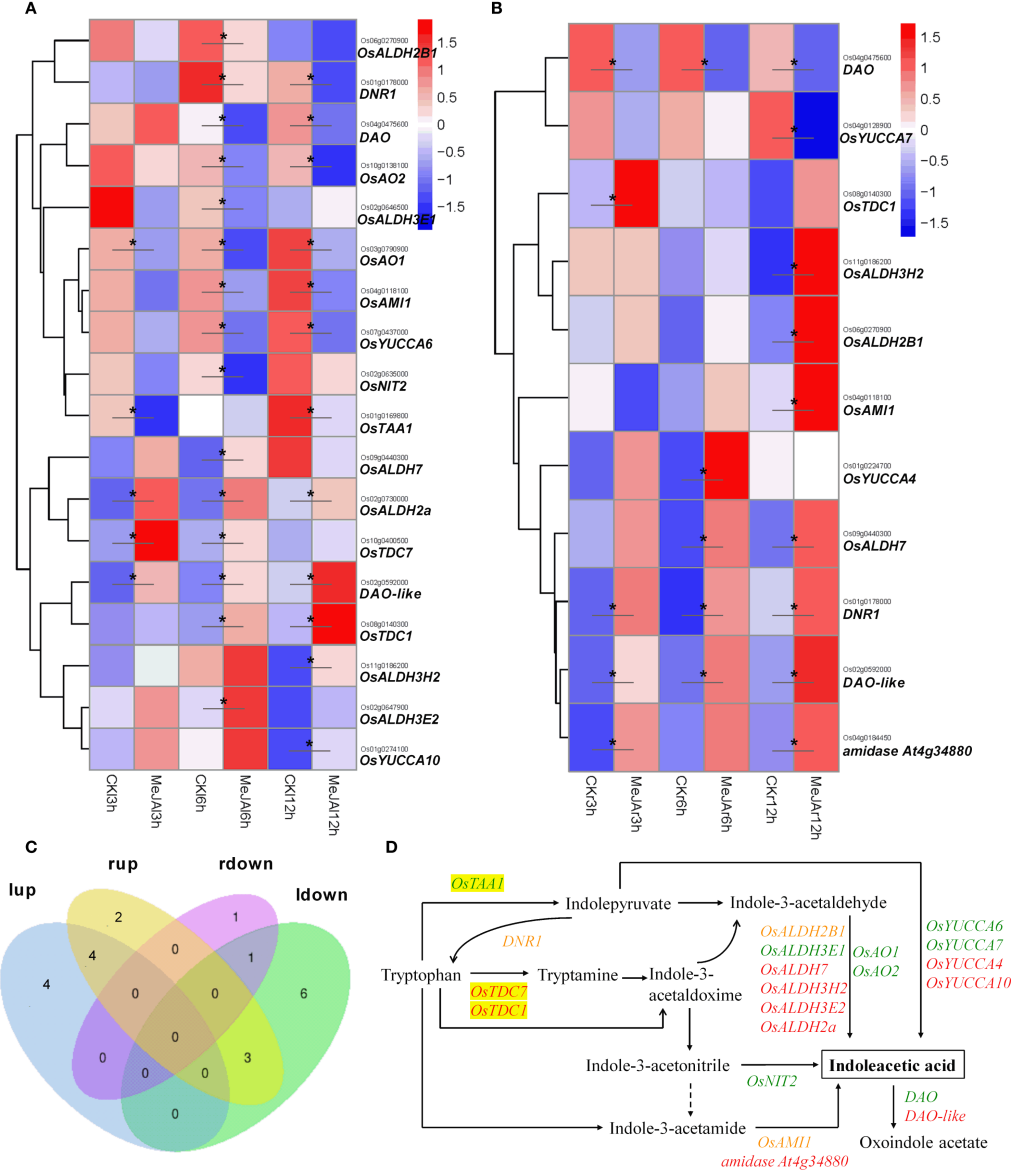
RNA-seq showing effect of exogenous MeJA on the expression of genes associated with auxin synthesis/catabolism in the leaves and roots of rice plants. Hierarchical clustering was performed on DEGs involved in auxin synthesis/catabolism, utilizing FPKM values from both MeJA-treated and untreated rice leaves **(A)** and roots **(B)**. CK, Control group. **(C)** Venn diagram that illustrates the overlap of up-regulated and down-regulated DEGs in the leaves and roots of MeJA-treated rice. The terms ‘lup’ and ‘rup’ refer to up-regulated genes in leaves and roots, respectively, while ‘ldown’ and “rdown”denotes down-regulated genes in the leaves and roots, respectively. **(D)** DEGs within the auxin synthesis/catabolism, with red, green and yellow markers indicating up-regulated, down-regulated, and both up- and down-regulated genes in the leaves or roots of MeJA-treated rice. Data were obtained from three biological replicates. DEGs were identified based on a fold change > 1.5 compared to the control, with a q-value (p-adjusted) < 0.05 (marked with *).

In the leaves, treatment with MeJA significantly up-regulated the expression of seven genes related to auxin synthesis, including the indole-3-pyruvate monooxygenase gene *YUCCA10*, which is involved in the IPA pathway; the tryptophan decarboxylase genes *OsTDC1* and *OsTDC7*, and the aldehyde dehydrogenase genes *OsALDH7*, *OsALDH3H2*, *OsALDH3E2*, and *OsALDH2a*, all of which are involved in the TAM pathway. Additionally, one auxin-catabolizing gene, *DIOXYGENASE FOR AUXIN OXIDATION* (*DAO-like*) *Os02g0592000*, was also significantly up-regulated at all timepoints (3h/6h/12h) post-treatment. Conversely, within a 12-hour period, MeJA treatment significantly down-regulated the expression of nine auxin synthesis-related genes: the tryptophan aminotransferase gene *OsTAA1*(*FIB*/*OsTAR2*), the amino transferase gene *DNR1*, and the monooxygenase gene *OsYUCCA6*, all involved in the IPA pathway; the aldehyde dehydrogenase genes *OsALDH2B1* and *OsALDH3E1*, and the indole-3-acetaldehyde oxidase genes *OsAO1* and *OsAO2*, involved in the TAM pathway; the nitrilase gene *OsNIT2*, involved in the IAOx pathway; and the amidase gene *OsAMI1*, involved in the IAM pathway. Furthermore, one auxin-catabolizing gene, *DAO Os04g0475600*, showed slightly upregulated at 3h but was significantly down-regulated at 6h and 12h post-treatment (**Figures 4A, C, D**).

In the roots, exogenous MeJA treatment significantly up-regulated the expression of eight genes associated with auxin synthesis: *DNR1* and *OsYUCCA4*, which are involved in the IPA pathway; *OsTDC1*, *OsALDH7*, *OsALDH2B1*, and *OsALDH3H2*, which are part of the TAM pathway; and *OsAMI1* and *amidase At4g34880*, which are involved in the IAM pathway. Additionally, one auxin-catabolizing gene, *DAO-like Os02g0592000*, was significantly up-regulated. On the other hand, the expression of one auxin synthesis-related gene, *OsYUCCA7*, involved in the IPA pathway, and one auxin-catabolizing gene, *DAO Os04g0475600*, was significantly down-regulated by MeJA-treatment (**Figures 4B–D**).

In both leaves and roots, the expression of genes *OsTDC1*, *OsALDH7*, *OsALDH3H2*, and *DAO-like Os02g0592000* was significantly up-regulated, whereas the expression of gene *DAO Os04g0475600* was significantly down-regulated. Notably, the expression of genes *DNR1*, *OsALDH2B1*, and *OsAMI1* was significantly down-regulated in leaves but up-regulated in roots (**Figures 4A–C**). The findings indicate that three IAA synthesis genes (*OsTDC1*, *OsALDH7*, *OsALDH3H2*) exhibit significant upregulation in both tissues (though at distinct time points), while other genes show tissue-divergent responses. Specifically, *OsTDC1* was induced at 6-12h in leaves and 3h in roots; *OsALDH7* was up-regulated at 6h in leaves and 6-12h in roots; and *OsALDH3H2* showed increased expression at 12h in both tissues. This demonstrates that JA signaling predominantly induces site- and time-dependent changes in IAA synthesis genes.

### 3.5 Changes in expression profiling of auxin signaling transduction/transport related genes

Auxin perception and signal transduction are intricately linked to the transcriptional regulation of downstream genes through mechanisms involving 26S proteasome-dependent protein degradation (Pérez and Goossens, 2013; Gallei et al., 2020). In the absence of auxin, AUXIN/INDOLE-3-ACETIC ACID (AUX/IAA) transcriptional repressor proteins interact directly with TOPLESS (TPL) corepressors to inhibit the activity of ARFs at the promoter regions of auxin-responsive genes. Upon increased auxin levels, where the IAA is the bioactive form, the recruitment of AUX/IAA by the Skp1-Cullin-F-box (SCF) E3 ubiquitin ligase complex, which includes the F-box protein TRANSPORT INHIBITOR RESPONSE 1 (SCF^TIR1^), facilitates the ubiquitination and subsequent degradation of AUX/IAA. This degradation process releases ARFs, enabling the transcription of auxin-responsive genes. These genes are categorized into three major families: Aux/IAA, auxin-inducible amidosynthetase genes such as Gretchen Hagen 3 (GH3), and Small Auxin Up RNA (SAUR). Additionally, the level of auxin in specific tissues and cells is regulated by its polar transport. Studies proposed that auxin enters plant cells via AUXIN1 (AUX1)/LIKE AUX1 (LAX) influx carriers and exits cells through efflux carriers belonging to the PIN-FORMED (PIN) and P-GLYCOPROTEIN (PGP) ABC transporter families (Petrásek and Friml, 2009; Gomes and Scortecci, 2021).

**Figure 5:**
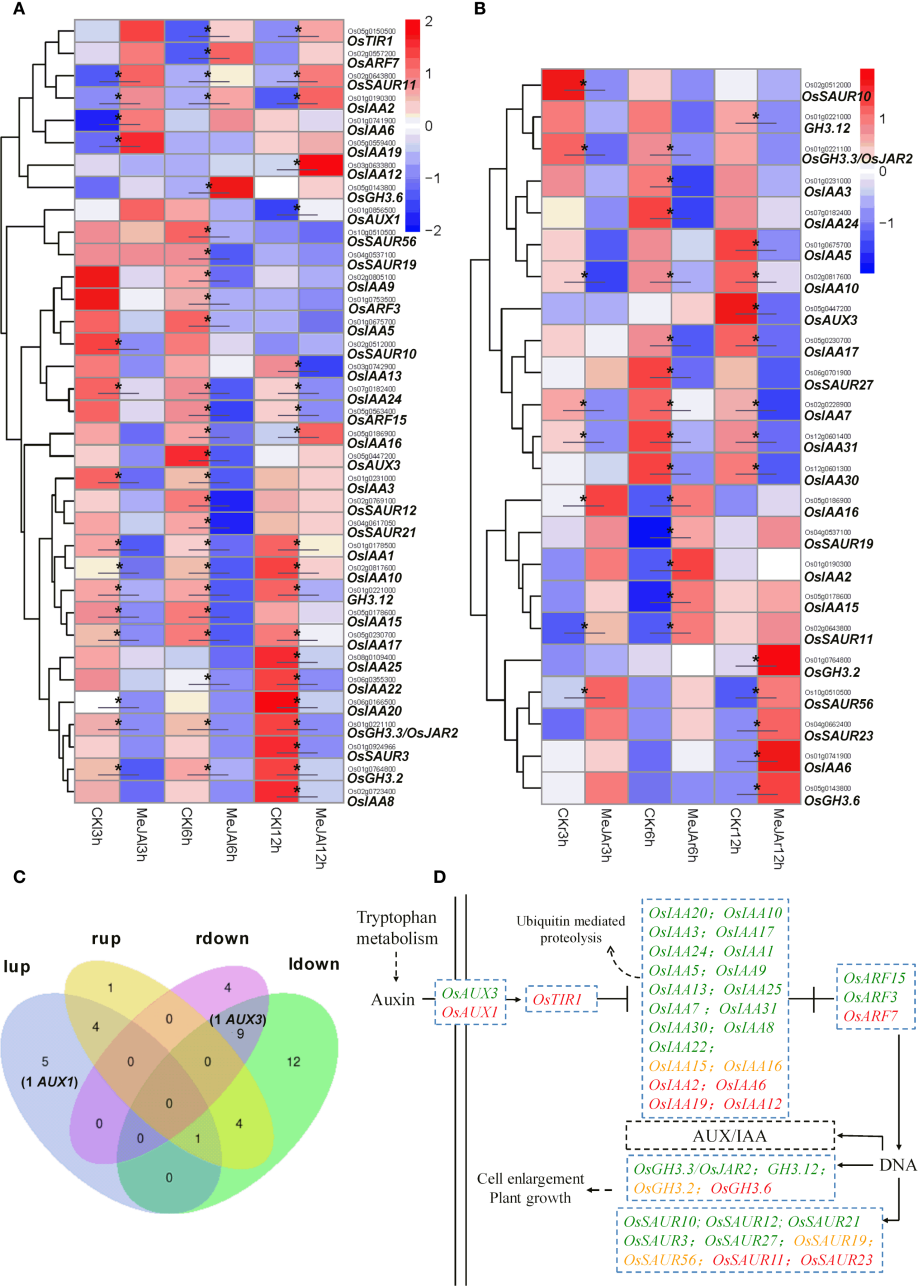
RNA-seq showing effect of exogenous MeJA on the expression of genes associated with auxin signaling transduction/influx transport in the leaves and roots of rice plants. Hierarchical clustering was performed on DEGs involved in auxin signaling/influx transport, utilizing FPKM values from both MeJA-treated and untreated rice leaves **(A)** and roots **(B)**. CK, Control group. **(C)** Venn diagram that illustrates the overlap of up-regulated and down-regulated DEGs in the leaves and roots of MeJA-treated rice. The terms ‘lup’ and ‘rup’ refer to up-regulated genes in leaves and roots, respectively, while ‘ldown’ and “rdown”denotes down-regulated genes in the leaves and roots, respectively. **(D)** DEGs within the auxin signaling/influx transport, with red, green and yellow markers indicating up-regulated, down-regulated, and both up- and down-regulated genes in the leaves or roots of MeJA-treated rice. Data were obtained from three biological replicates. DEGs were identified based on a fold change > 1.5 compared to the control, with a q-value (p-adjusted) < 0.05 (marked with *).

In the leaves, within a 12-hour period, MeJA treatment resulted in a significant up-regulation of eight genes associated with the auxin signaling pathway. These include the auxin receptor *OsTIR1*, four members of the *Aux/IAA* gene family (*OsIAA2*, *OsIAA6*, *OsIAA19*, and *OsIAA12*), as well as *OsARF7*, *OsGH3.6*, and *OsSAUR11*. Conversely, MeJA treatment significantly down-regulated the expression of twenty-four genes related to the auxin signaling pathway. This group comprises thirteen members of the *Aux/IAA* gene family (*OsIAA20*, *OsIAA10*, *OsIAA3*, *OsIAA17*, *OsIAA24*, *OsIAA15*, *OsIAA1*, *OsIAA5*, *OsIAA9*, *OsIAA13*, *OsIAA25*, *OsIAA22*, and *OsIAA8*), two auxin response factors (*OsARF15* and *OsARF3*), three members of the *GH3* gene family (*OsGH3.3/OsJAR2*, *OsGH3.2*, and *GH3.12*), and six members of the *SAUR* gene family (*OsSAUR10*, *OsSAUR19*, *OsSAUR12*, *OsSAUR56*, *OsSAUR21*, and *OsSAUR3*). Notably, the expression of the Aux/IAA gene *OsIAA16* was initially down-regulated at 6 hr post-treatment, followed by a significant up-regulation at 12 hr (**Figures 5A, C, D**).

In the roots, treatment with MeJA led to a significant up-regulation in the expression of ten genes associated with the auxin signaling pathway. This includes four members of the *Aux/IAA* gene family (*OsIAA*2, *OsIAA6*, *OsIAA15*, and *OsIAA16*), two members of the *GH3* gene family (*OsGH3.6* and *OsGH3.2*), and four members of the *SAUR* gene family (*OsSAUR11*, *OsSAUR19*, *OsSAUR56*, and *OsSAUR23*). Conversely, the expression of twelve auxin signaling pathway-related genes was significantly down-regulated, comprising eight members of the *Aux/IAA* gene family (*OsIAA10*, *OsIAA3*, *OsIAA17*, *OsIAA24*, *OsIAA5*, *OsIAA7*, *OsIAA3*, and *OsIAA30*), two *GH3* genes (*OsGH3.3/OsJAR2* and *GH3.12*), and two SAUR genes (*OsSAUR10* and *OsSAUR27*) (**Figures 5B–D**). In both leaves and roots, the expression of *OsIAA2*, *OsIAA6*, *OsGH3.6*, and *OsSAUR11* was significantly up-regulated. In contrast, the expression of *OsIAA10*, *OsIAA3*, *OsIAA17*, *OsIAA24*, *OsIAA5*, *OsGH3.3/OsJAR2*, *GH3.12*, and *OsSAUR10* was down-regulated. Notably, the expression of *OsIAA15*, *OsGH3.2*, *OsSAUR19*, and *OsSAUR56* was significantly down-regulated in leaves but up-regulated in roots (**Figure 5**).

**Figure 6:**
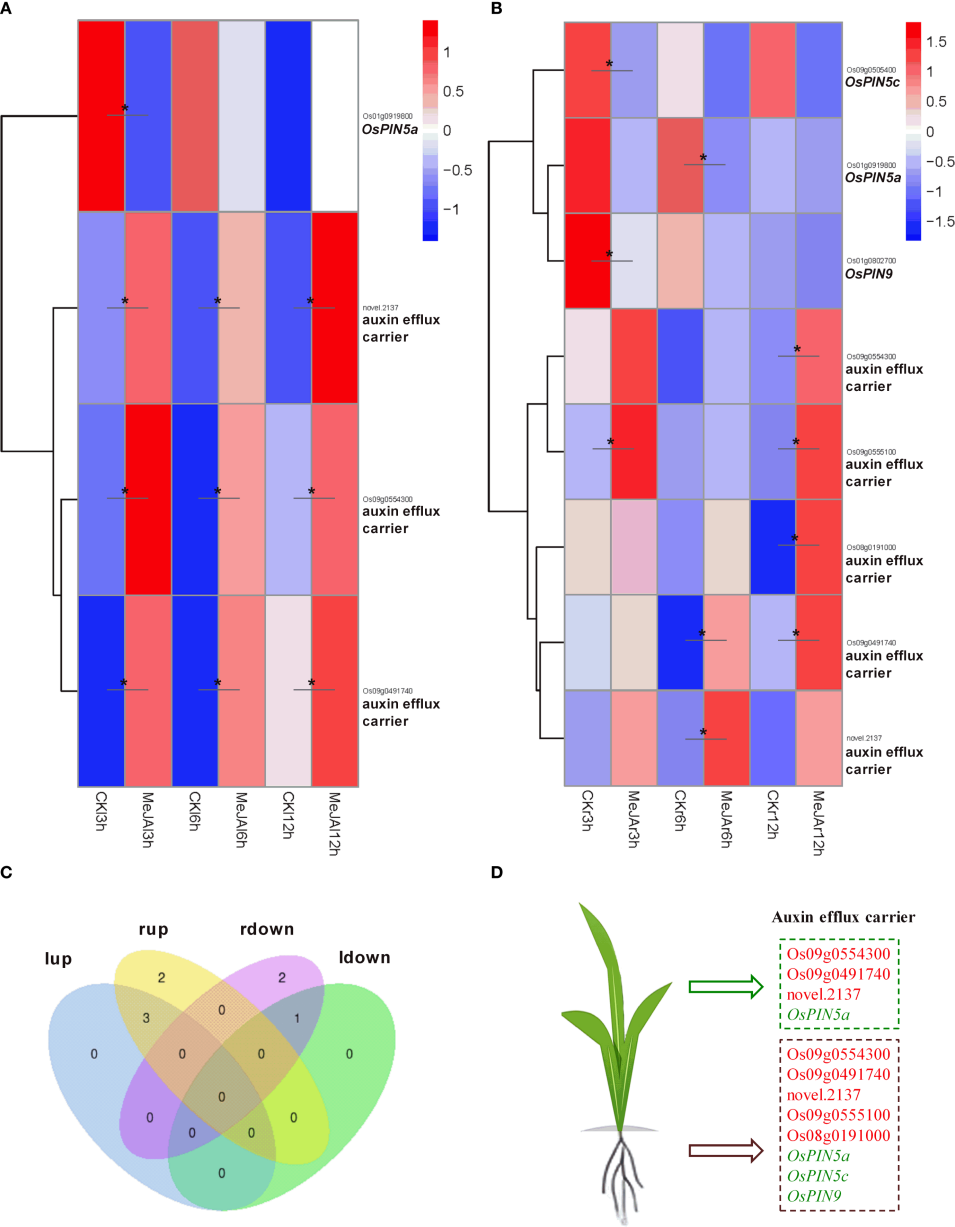
RNA-seq showing effect of exogenous MeJA on the expression of genes associated with auxin efflux transport in the leaves and roots of rice plants. Hierarchical clustering was performed on DEGs involved in auxin efflux transport, utilizing FPKM values from both MeJA-treated and untreated rice leaves **(A)** and roots **(B)**. CK, Control group. **(C)** Venn diagram that illustrates the overlap of up-regulated and down-regulated DEGs in the leaves and roots of MeJA-treated rice. The terms ‘lup’ and ‘rup’ refer to up-regulated genes in leaves and roots, respectively, while ‘ldown’ and “rdown”denotes down-regulated genes in the leaves and roots, respectively. **(D)** DEGs within the auxin efflux transport, with red and green markers indicating up-regulated and down-regulated genes in the leaves or roots of MeJA-treated rice. Data were obtained from three biological replicates. DEGs were identified based on a fold change > 1.5 compared to the control, with a q-value (p-adjusted) < 0.05 (marked with *).

MeJA treatment resulted in a significant up-regulation of the expression of the auxin influx carrier gene *OsAUX1* in the leaves, whereas it caused a significant down-regulation of *OsAUX3* expression in both leaves and roots (**Figure 5**). Regarding the auxin efflux carriers, MeJA treatment notably up-regulated the expression of three efflux carrier genes: *novel.2137*, *Os09g0554300*, and *Os09g0491740*, while significantly down-regulating *OsPIN5a* expression in the leaves. Additionally, in the roots, MeJA treatment significantly up-regulated the expression of five efflux carrier genes: *Os09g0554300*, *Os09g0555100*, *Os08g0191000*, *Os09g0491740*, and *novel.2137*, and significantly down-regulated the expression of *OsPIN5a*, *OsPIN5c*, and *OsPIN9*. In both leaves and roots, the expression of *novel.2137*, *Os09g0554300*, and *Os09g0491740* was significantly up-regulated, whereas *OsPIN5a* expression was significantly down-regulated (**Figure 6**).

### 3.6 Combined action of hormones on rice plant length, weight, and leaf senescence

**Figure 7:**
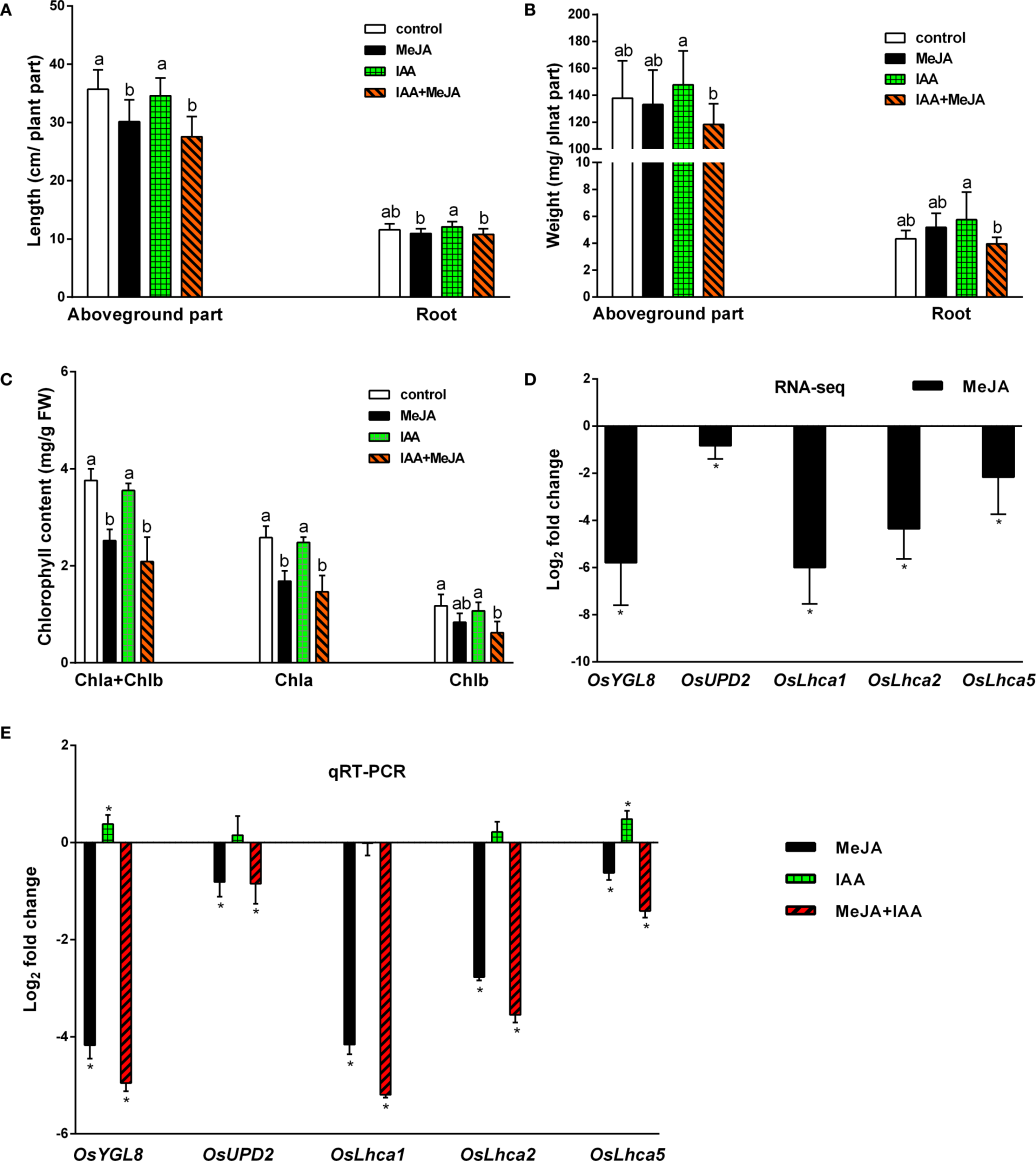
Effects of exogenous application of MeJA and IAA on plant length, weight, and leaf senescence of rice plants. **(A)** Lengths of aboveground parts and roots. Values are mean ± standard SD (n = 12). **(B)** Lengths of aboveground parts and roots. Values are mean ± standard SD (n = 12). **(C)** Chlorophyll contents of leaves Values are mean ± SD (n = 4). **(D, E)** Relative transcript levels of five genes involved in chlorophyll biosynthesis from transcriptome analysis (RNA-seq) and quantitative real-time PCR analysis (qRT-PCR), respectively. Values are mean ± SD (n = 3). Fold change represents the gene expression ratio between the hormone-treated group and the control group (MeJA, MeJA: control; IAA, IAA: control; MeJA+IAA, MeJA+IAA: control). The chlorophyll biosynthesis associated genes examined include *OsYGL8*, encoding magnesium-protoporphyrin IX monomethyl ester oxidative cyclase; *OsUPD2*, encoding uroporphyrinogen decarboxylase 2; and *OsLhca1*, *OsLhca2*, and *OsLhca5*, encoding light-harvesting complex (LHC) chlorophyll a/b-binding proteins. Significant differences among treatments are indicated by letters above the bars (Tukey’s multiple range test, P < 0.05). Asterisks denote significant differences between hormone-treated groups and control groups, as determined by RNA-seq (q-value, p-adjusted < 0.05) and qRT-PCR (Tukey’s multiple range test, *p <*0.05).

Previous research has indicated that JAs are implicated in various developmental processes, including growth inhibition and the induction of leaf senescence, whereas auxins are vital plant growth regulators and play a role in suppressing leaf senescence (Jiang et al., 2014). Given that MeJA treatment significantly reduced IAA levels in rice plants (**Figure 3B**), we investigated whether exogenous IAA could counteract the effects of MeJA on growth inhibition and leaf senescence induction. After a seven-day treatment period, MeJA was observed to exert a pronounced inhibitory effect on the length of the aboveground parts of rice plants; the application of 0.1 μM IAA did not promote their growth, nor did it alleviate the effect induced by MeJA (**Figure 7A**). Treatments with MeJA, IAA, and the combination of MeJA and IAA did not significantly alter root length or the biomass of either the aboveground parts or the roots (**Figures 7A, B**). However, unexpectedly, the combined MeJA and IAA treatment resulted in the lowest measurements for aboveground length, root length, and biomass of both the aboveground parts and roots compared to the control, MeJA, and IAA groups. MeJA treatment significantly reduced rice leaf chlorophyll content. 0.1 μM IAA applications neither altered chlorophyll levels alone nor reversed MeJA-induced reduction in MeJA+IAA co-treated plants (**Figure 7C**). Transcriptome analysis indicated that the expression of five genes associated with chlorophyll synthesis (*OsYGL8*, *OsUPD2*, *OsLhca1*, *OsLhca2*, and *OsLhca5*) was significantly down-regulated by MeJA (**Figure 7D**). These findings were corroborated by qRT-PCR (**Figure 7E**), which also confirmed the accuracy of the Illumina sequencing results. qRT-PCR analysis further demonstrated that IAA treatment significantly up-regulated the expression of *OsYGL8*, *OsLhca2*, and *OsLhca5*, but did not significantly alter the expression of *OsUPD2* and *OsLhca1*. The combined treatment of MeJA and IAA led to a significant down-regulation of these genes compared to the control group, with a more pronounced decline in gene expression observed in the MeJA+IAA group than in the MeJA-only treatment group (**Figure 7E**). Overall, these results suggest that the MeJA and IAA co-treatment exhibited an additive negative effect on plant growth, rather than demonstrating an antagonistic action of auxin against the growth inhibition induced by JAs, yet the underlying mechanisms driving this additive interaction remains to be elucidated.

To further validate the transcriptomic results, we performed qPCR analysis on rice leaves treated with a lower concentration of MeJA (80 μM). The results revealed that the expression of *OASB2*, *OsTSA1*, and *OsTSB2* was up-regulated at 6 hours, while that of *OsTAA1* and *OsGH3.2* was down-regulated after 3 hours (**Supplementary Figure S4**). These trends are consistent with the RNA-seq data obtained under 500 μM MeJA (**Figures 2A**, **4A**, **5A**). The high consistency between the qPCR and RNA-seq results supports the reliability of our transcriptomic profiling.

## 4 Discussion

The JA signaling pathway exhibits notable similarities to the auxin signaling pathway. Under normal growth conditions, when JA concentrations are low, JA-responsive genes are repressed by JA ZIM-DOMAIN (JAZ) proteins, which physically interact with various downstream transcription factors (TFs); Upon the perception of bioactive JA, the JA receptor CORONATINE INSENSITIVE1 (COI1), a component of the SCF^COI1^ E3 ligase complex, facilitates the ubiquitination and subsequent degradation of JAZ proteins via the 26S proteasome (Wasternack and Hause, 2013; Huang et al., 2017). This degradation releases the TFs, thereby enabling the expression of JA-responsive genes and the initiation of JA-mediated responses. Consequently, JA-mediated transcriptional responses are intricately linked with the accumulation of JAs, particularly the bioactive form. JA-Ile has been recognized as the sole bioactive form of the hormone to date (Fonseca et al., 2009; Li et al., 2022). In addition to JA-Ile, other forms of JAs, including JA and MeJA, do not have activity to trigger the COI1-JAZ interaction. Instead, they serve as precursors that are converted into JA-Ile in response to specific environmental cues, thereby becoming effective signaling molecules (Li et al., 2022). An optimal concentration of JA-Ile is crucial, as excessive accumulation can lead to overactivation of the defense mechanisms, which subsequently compromises plant growth. In this study, rice leaves treated with MeJA to simulate environmental stress exhibited a strongly increase in the accumulation of JA-Ile, JA, MeJA, and DJA (**Figure 1**), indicating a robust activation of the defense response in the leaves. Conversely, in the roots, MeJA treatment of the leaves resulted in elevated levels of JA, MeJA, and DJA, but did not alter the JA-Ile concentration, suggesting that the defense response in the roots remained in a standby mode rather than being triggered. These findings imply that JA signaling may regulate site-specific defense responses to prevent excessive defense activation, thereby optimizing plant fitness under stress conditions.

Previous research has demonstrated that MeJA treatment enhances the production of active auxin, specifically free IAA, in *Arabidopsis* plants (Dombrecht et al., 2007; Sun et al., 2009; Hentrich et al., 2013). The genes *ASA1* and *ASB1*, which encode anthranilate synthase responsible for catalyzing the conversion of chorismate to anthranilate—the first rate-limiting step in the Trp biosynthetic pathway—have been implicated in mediating JA-induced IAA accumulation and transport. Additionally, the monooxygenase genes *YUC2*, *YUC8*, and *YUC9* may also participate in JA-regulated auxin biosynthesis, as their expression is upregulated by MeJA (Sun et al., 2009; Hentrich et al., 2013; Cai et al., 2014). In this study, transcriptome sequencing indicated that MeJA indeed augmented the metabolic flux into the Trp pathway by significantly upregulating the expression of 17 genes involved in Trp biosynthesis, including three anthranilate synthase genes: *OASA2*, *OASB1*, and *OASB2* (**Figure 2**). However, contrary to the previous findings, our study showed that JA signaling significantly reduced IAA content in both leaves and roots of rice plants, while significantly increasing the content of ICA and ICAld in rice leaves (**Figures 3B, D, E**). The IPA pathway has been identified as the primary mechanism for IAA synthesis in plants, where Trp is initially converted to IPA by the TAA family of aminotransferases, followed by the conversion of IPA to IAA by the YUC family of flavin mono-oxygenases (Cao et al., 2019; Gomes and Scortecci, 2021). Transcriptome sequencing analysis revealed that MeJA treatment significantly down-regulated the expression of the Trp aminotransferase gene *OsTAA1* (*FIB*) and the monooxygenase gene *OsYUCCA6*, both associated with the IPA pathway, in the leaves of rice plants (**Figures 4A, D**). Yoshikawa et al. (2014) demonstrated that the loss of *OsTAA1* (*FIB*) function resulted in pleiotropic auxin deficiency phenotypes in rice, including abnormal leaves and vascular, small panicles, abnormal organ identity, and root development defects, along with a reduction in internal IAA levels, indicating that the *OsTAA1* gene plays a crucial role in IAA biosynthesis. Moreover, since the *OsTAA1* responsible for the initial conversion of Trp to IPA, the down-regulation of *OsTAA1* indicated the reduction of Trp flux into the IPA pathway in the leaves. On the other hand, MeJA treatment significantly up-regulated the *DNR1* expression and down-regulated *OsYUCCA7* expression, which also involved in the IPA pathway, in the roots of rice plants (**Figures 4B, D**). The *DNR1* gene encodes an aminotransferase that converts IPA back to Trp, thereby restricting the synthesis of IAA; a loss-of-function mutation at the *DNR1* locus is associated with enhanced nitrogen uptake and assimilation, leading to improved rice yield (Zhang et al., 2021). Collectively, these results suggest that JA signaling reduces IAA levels in rice, at least partly due to the suppression of the major IAA biosynthesis pathway–IPA pathway. This suppression is evidenced by the down-regulation of *OsTAA1* and *OsYUCCA6* expression in the leaves, as well as the up-regulated *DNR1* expression and down-regulated *OsYUCCA7* expression in the roots. Additionally, JA signaling was found to up-regulate *OsYUCCA10* expression while down-regulating *DNR1* expression in the leaves, and to up-regulate *OsYUCCA4* expression in the roots. These findings indicate that the regulation of the IPA pathway by JA signaling is complex, with these genes likely playing a role in IAA synthesis in rice during JA-mediated stress responses.

Tryptophan decarboxylase (TDC) is a cytosolic enzyme responsible for converting Trp to IAA precursor TAM, thereby catalyzing the initial step of the TAM pathway for IAA biosynthesis (Kang et al., 2009). Treatment with MeJA significantly up-regulated the expression of *OsTDC7* in the leaves and *OsTDC1* in both leaves and roots of rice plants (**Figure 4**), indicating that JA signaling substantially enhances Trp flux into the TAM pathway in rice. However, the increased expression of *OsTDC7* and *OsTDC1* did not lead to elevated levels of IAA in rice leaves and roots; instead, it showed a reduction of IAA content (**Figure 3B**). This result is likely attributable to three factors described below. (i) Our findings indicate that JA signaling further mediates IAA degradation. MeJA treatment significantly reduced IAA levels while elevating ICA and ICAld accumulation in rice leaves (**Figures 3B, D, E**). This aligns with JA-induced up-regulation of peroxidases (PODs) (**Supplementary Figure S2**; for methods, see Wu et al., 2019), enzymes implicated in IAA decarboxylation to produce ICA and ICAld as intermediates or end products (Sagee et al., 1989; Normanly, 1997). It is worth noting that exogenous IAA exacerbated MeJA-induced leaf senescence (**Figures 7C–E**), indicating that JA signaling is likely to accelerate IAA catabolism. The resultant ICA and ICAld, known phytoalexin precursors (Que et al., 2022; Lu et al., 2024), synergized with MeJA to promote senescence (**Supplementary Figure S3**), mirroring ethylene-induced ICA-glu accumulation in senescing citrus leaves (Sagee et al., 1989). Concurrently, JA significantly up-regulated the *DAO-like* gene *Os02g0592000* (8.3 to 23.5-fold in leaves; 6.3 to 10.8-fold in roots; **Figures 4A, B, D**), which catalyzes IAA-to-oxIAA conversion. Thus, JA likely depletes free IAA via dual mechanisms: POD-mediated decarboxylation and DAO-dependent oxidation. (ii) An alternative pathway may exist that competes with the conversion of TAM to IAA under JA-mediated stress responses in rice. Both IAA and defensive indole glucosinolates (IGs)/camalexin (CL) share IAOx as a biosynthetic node via the same Trp pathway (Hansen and Halkier, 2005). There are two distinct pathways involving the *CYP79B* or *YUCCA* genes may contribute to IAOx synthesis (Zhao et al., 2001; Sugawara et al., 2009). In rice, lacking CYP79B orthologs, we propose YUCCA enzymes (e.g., OsYUCCA4/10, **Figure 4D**) mediate TAM-to-IAOx conversion via N-hydroxytryptamine (HTAM) (Zhao et al., 2001). JAs known induction of IGs in other plants (Schweizer et al., 2013; Zhang et al., 2016; Yi et al., 2022), suggesting a conserved strategy: under stress, JA signaling prioritizes Trp allocation to IAOx-derived defense compounds over IAA synthesis. While IAOx and IGs remain undetected in rice, their absence in prior studies may reflect methodological gaps—e.g., analyses under non-stressed conditions where IAOx pools are transient. This metabolic competition could explain why increased TAM flux fails to restore IAA levels during JA responses. Finally, this could be due to (iii) the JA-mediated suppression of the IPA pathway or the complex changes in gene expression downstream of the TAM pathway induced by JA signaling (**Figure 4**), even when the increased flux of Try into the TAM pathway is insufficient to maintain IAA levels in rice. These mechanisms collectively enhance JA-mediated defenses while accelerating senescence. Future work should clarify IAOx dynamics in stressed rice and elucidate how ICA/ICAld crosstalk with senescence pathways.

Plant growth, development, and stress response are regulated by a complex network of signaling pathways orchestrated by various phytohormones. Transcriptome analysis demonstrated that JA signaling induced extensive transcriptional changes in the regulation of auxin signaling transduction in rice (**Figure 5**). It is worth noting that, MeJA treatment in rice leaves resulted in significant alterations in the gene expression of eighteen *Aux/IAA* transcriptional repressors, with thirteen genes down-regulated, four genes up-regulated, and one gene initially down-regulated followed by up-regulation. In the roots, twelve *Aux/IAA* genes exhibited significant changes, with eight genes down-regulated and four up-regulated. These findings suggest that JA signaling predominantly down-regulates the expression of *Aux/IAA* repressor genes in the auxin signaling transduction, thereby derepressing ARFs and enhancing the auxin signaling response. In addition to biosynthesis and catabolism, conjugation to amino acids is a common mechanism for regulating hormone levels by converting active signaling forms into inactive forms for storage, transport, or degradation. The formation of indole-3-acetic acid-amino acid (IAA-aa) conjugates is catalyzed by a group of enzymes belonging to the GH3 family (Zhang et al., 2016; Casanova-Sáez et al., 2021). The application of MeJA significantly altered the expression of four *GH3* genes in both leaves and roots. Specifically, in the leaves, three genes were down-regulated while one was up-regulated; in the roots, two genes were down-regulated and two were up-regulated. The down-regulation of *GH3* gene expression suggests a reduction in the formation of IAA-aa conjugates catalyzed by these GH3 members, which may help maintain the levels of bioactive free IAA in rice under JA response conditions. Notably, OsGH3.3/OsJAR2, previously identified as a JA-Ile synthase (Wakuta et al., 2011), was also down-regulated, potentially inducing a negative feedback loop in JA signaling by decreasing the levels of bioactive JA-Ile in rice. Furthermore, MeJA treatment significantly up-regulated the expression of the auxin receptor gene *OsTIR1* in rice leaves. Collectively, these findings suggest that JA signaling orchestrates transcriptional regulation to enhance auxin transduction and response in rice, particularly in the leaves, by down-regulating multiple *Aux/IAA* repressors and *GH3* members, while up-regulating the auxin receptor *OsTIR1*. This regulatory mechanism may allow rice to fine-tune its growth in response to stress signals under MeJA-mediated conditions of reduced IAA levels.

Additionally, JA signaling was found to mediate alterations in the gene expression of *nitrilase OsNIT2*, *amidase OsAMI1*, *At4g34880*, and *DAO Os04g0475600*, which are associated with IAA synthesis and catabolism. It also affected the expression of several *SAUR* genes and *ARFs* related to auxin signaling transduction, as well as genes involved in auxin transport (refer to the Results section for details). The physiological implications of these gene expression changes in response to JA signaling warrant further investigation. Notably, JA signaling did not significantly affect the levels of IBA in either the leaves or roots of rice (see **Figure 3C**). IBA serves as a precursor molecule, potentially acting as a storage form of IAA. Moreover, IBA may either trigger plant responses independently of IAA or be converted into IAA, thereby contributing to IAA’s role in plant development and stress response (Gomes and Scortecci, 2021). The stability of IBA levels following MeJA treatment in rice suggests that IBA is crucial for maintaining plant development under stress conditions.

**Figure 8:**
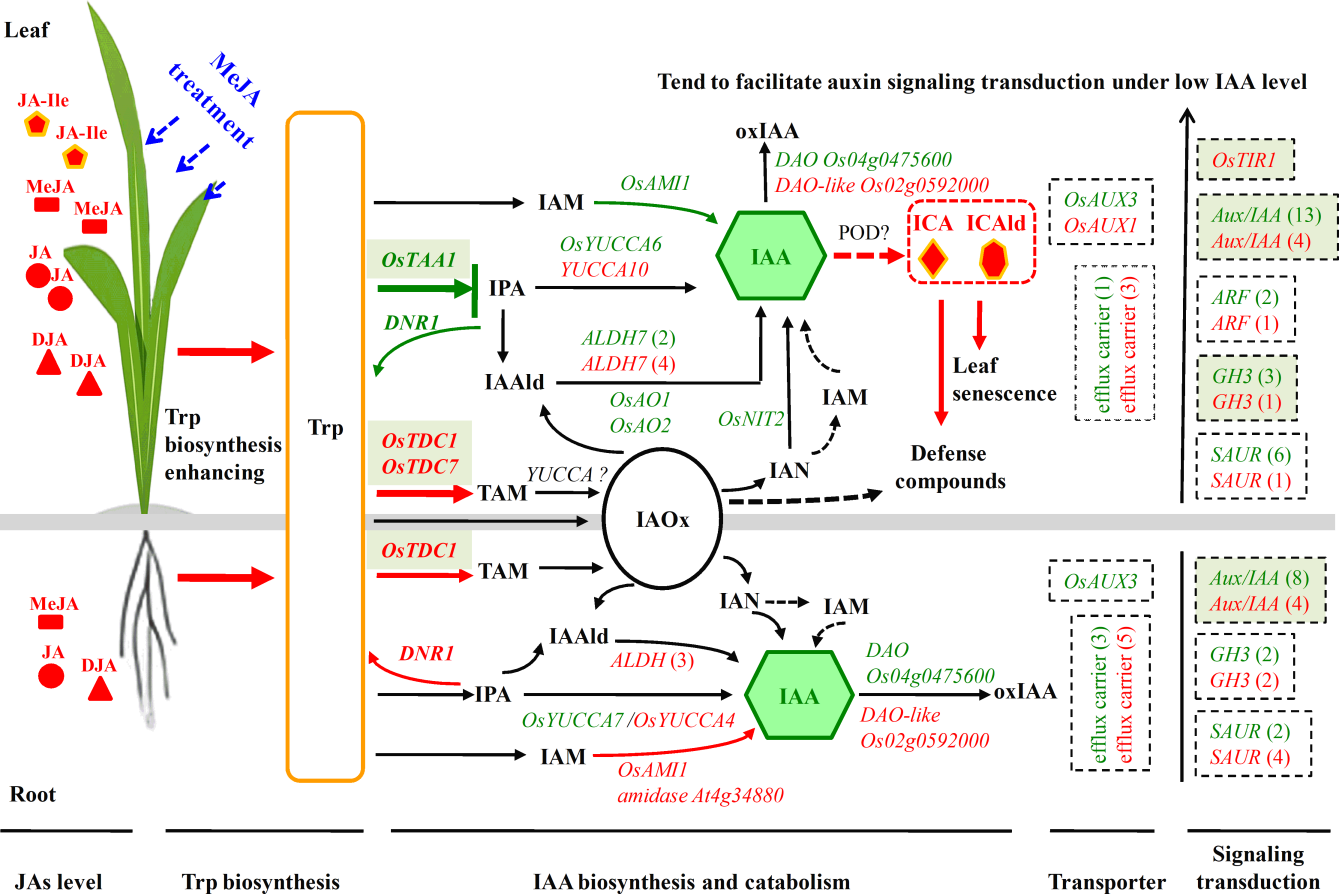
A conceptual model is proposed to elucidate the potential roles of JA signaling in modulating auxin levels and transcriptional regulation of auxin metabolism, transport, and signaling. In this model, red and green markings indicate up-regulated and down-regulated hormones or genes in MeJA-treated rice compared to the control group. Solid arrows represent direct causal relationships, while dashed arrows suggest probable causal links.

In conclusion, JA signaling significantly impacted the profiles of JAs and auxins, while also orchestrate large-scale changes in the transcriptional regulation of Trp biosynthesis, as well as IAA biosynthesis, catabolism, signaling transduction, and transport in rice leaves and roots, as illustrated in the proposed conceptual model (**Figure 8**). These JA signaling-induced alterations exhibit distinct patterns between leaves and roots, with changes in leaves showing a more pronounced response to JA signaling than those in roots. Our findings reveal that JA signaling-mediated transcriptional regulation of the auxin metabolism and signaling transduction constitutes a complex yet precise regulatory network. A deeper understanding of these mechanisms may facilitate advancements in plant breeding and engineering strategies aimed at optimizing rice resilience and yield under stress conditions.

## Data Availability

The datasets presented in this study can be found in online repositories. The names of the repository/repositories and accession number(s) can be found below: https://www.ncbi.nlm.nih.gov/, PRJNA1299722.
