# Supplementary material for: Integration of transcriptome and phytohormone analyses reveals antagonistic interactions between jasmonate and auxin signaling pathways in rice (Oryza sativa L.)
Source: Front Plant Sci. 2025 Sep 17;16:1622785. doi: 10.3389/fpls.2025.1622785 (PMC12484194; doi:10.3389/fpls.2025.1622785)
Supplement: Supplementary file 1 [file DataSheet1.docx]

**Supporting information**

**Table S1 The quality control and mapping information of transcriptome analysis**

|  | **Sample** | **Q20 (%)** | **Q30 (%)** | **GC (%)** | **Unique-map (%)** |
| --- | --- | --- | --- | --- | --- |
| leaf | CK3h | 97.85±0.06 | 94.02±0.05 | 52.08±1.53 | 93.47±0.01 |
|  | MeJA3h | 97.94±0.28 | 94.25±0.78 | 51.33±1.56 | 94.17±00 |
|  | CK6h | 97.61±0.31 | 93.51±0.67 | 52.65±1.43 | 92.99±0.01 |
|  | MeJA6h | 97.88±0.06 | 93.97±0.13 | 50.29±0.24 | 93.87±0.00 |
|  | CK12h | 98.10±0.21 | 94.55±0.43 | 51.16±0.60 | 92.27±0.01 |
|  | MeJA12h | 97.30±0.22 | 93.06±0.32 | 50.54±0.43 | 90.75±0.01 |
| root | CK3h | 97.78±0.30 | 93.89±0.62 | 51.39±1.34 | 87.51±0.06 |
|  | MeJA3h | 97.27±0.62 | 92.58±1.59 | 51.35±1.84 | 84.18±0.06 |
|  | CK6h | 97.57±0.10 | 93.44±0.18 | 51.28±0.44 | 90.22±0.01 |
|  | MeJA6h | 97.54±0.23 | 93.41±0.32 | 51.07±1.43 | 83.74±0.06 |
|  | CK12h | 97.76±0.18 | 93.77±0.28 | 51.30±0.75 | 86.07±0.04 |
|  | MeJA12h | 97.66±0.57 | 93.42±1.50 | 52.45±1.23 | 87.99±0.01 |

**Table S2 Catalog numbers of phytohormone standards from ZZ Standards Co., Ltd**

| **Compound** | **Catalog Number** |
| --- | --- |
| IAA | 87-51-4 |
| IBA | 133-32-4 |
| ICA | 771-50-6 |
| ICAld | 487-89-8 |
| MeIAA | 1912-33-0 |
| JA | 77026-92-7 |
| MeJA | 39924-52-2 |
| JA-Ile | 120330-92-9 |
| DJA | 3572-64-3 |

**Table S3 Primers used in quantitative real-time PCR analysis**

| **Gene name** | **Forward primer (5’-3’)** | **Reverse primer (5’-3’)** | **Reference** |
| --- | --- | --- | --- |
| *OsActin* | TGGACAGGTTATCACCATTGGT | CCGCAGCTTCCATTCCTATG | Ye et al., 2013 |
| *OsYGL8* | TTCAACTTGGCACTGGACCT | CCTGGCACCAGTTCTCAAAG | * |
| *OsUPD2* | GCAAGTGGATCTGGTGGAC | AAAGAACGCCAGGGTCAACA | * |
| *OsLhca1* | TCTACCCTTGAGCTTGGCAC | CGCGCGCAATAATCAACCAA | * |
| *OsLhca2* | TGGTGGTCTGTGGTTTGACC | TGGAACCAAGCTCCCATGAC | * |
| *OsLhca5* | TGGCATCCTTGCGACTGATT | AAGAAGAGTGCCGTGGTGTT | * |

* designed by ourselves

Ye M, Song YY, Long J, Wang RL, Baerson SR, Pan ZQ, Zhu-Salzmand K, Xie JF, Cai KZ, Luo SM, Zeng RS. 2013. Priming of jasmonate-mediated antiherbivore defenseresponses in rice by silicon. Proceedings of the National Academy of Sciences, USA, 110(38): 3631-3639.


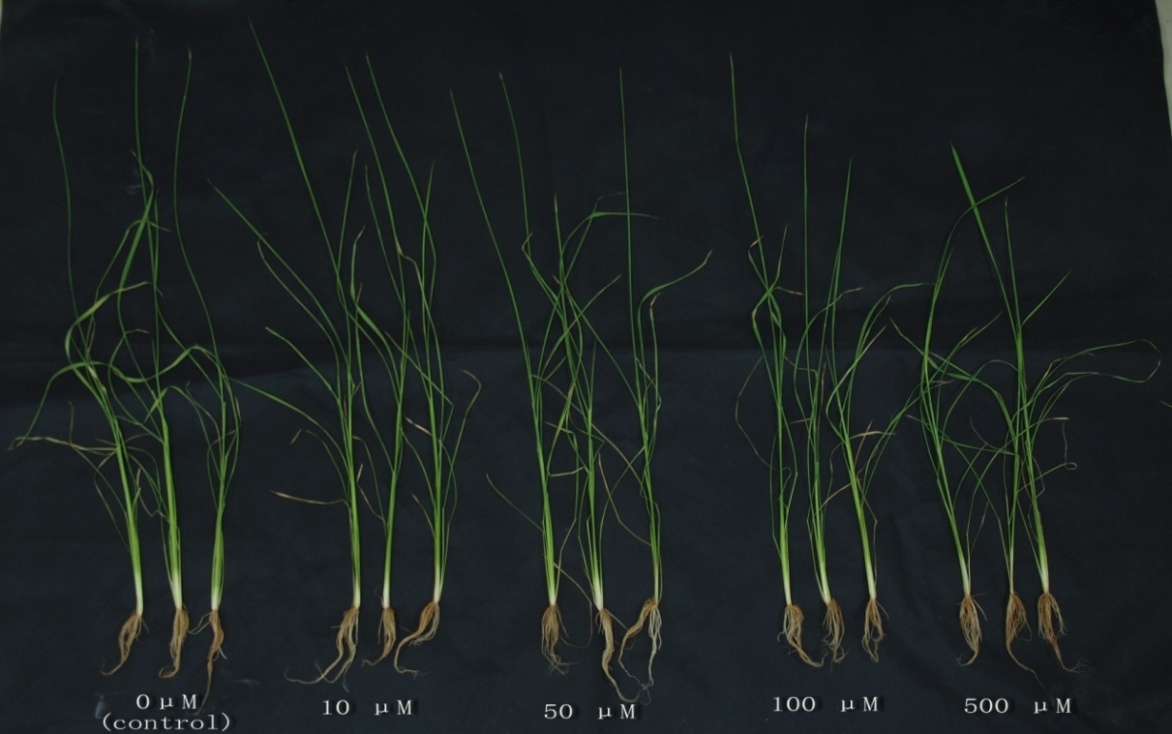


**Fig. S1 Growth phenotypes of rice seedlings under foliar MeJA treatment (15 days)**. Note:﹤100 μM show no significant suppression vs. control; 500 μM causes visible inhibition.





**Fig. S2** **Effect of exogenous MeJA on the relative abundance of peroxidase in the leaves and roots of rice plants.** The abscissa shows the logarithm of the protein ratio of two treatments (MeJA: control).



**Fig. S3 Effects of exogenous application of MeJA, ICA and ICAld on rice leaf senescence.** Rice leaves at the same developmental stage were placed into dishes containing distilled water supplemented with either water (control), 50 μM ICA, 50 μM ICAld, and/or 50 μM MeJA. The dishes were maintained under light or dark conditions at 26°C. The water or hormone solutions were replenished daily throughout the 72-hour experimental period.





**Fig. S4 Effects of 80 μM MeJA foliar spray treatment on gene expression in rice leave.** 3h and 6h indicate after 3 and 6 hours of MeJA treatment, respectively. Fold change represents the gene expression ratio between the MeJA-treated group and the control group. Values are mean ± SD (n = 3). Asterisks denote significant differences between MeJA-treated groups and control groups (Student’s t-test, *p*<0.05).
